# Supplementary material for: Control of brown adipose tissue adaptation to nutrient stress by the activin receptor ALK7
Source: eLife. 2020 May 5;9:e54721. doi: 10.7554/eLife.54721 (PMC7200161; doi:10.7554/eLife.54721)
Supplement: Supplementary file 1. — The PCR primers used in this study are listed here. [file elife-54721-supp1.docx]

**Supplementary Table 1: PCR primers**

| **Mouse genes** |  |  |
| --- | --- | --- |
| **Gene name** |  | **Sequence 5´to 3´** |
| *Adrb3* | Forward Primer | AGAAACGGCTCTCTGGCTTTG |
|  | Reverse Primer | TGGTTATGGTCTGTAGTCTCGG |
| *Alk7* | Forward Primer | GCTTTCCATAGCGAGTGGTC |
|  | Reverse Primer | ATCTCGGTGAGCAATAGCAG |
| *Alt1* | Forward Primer | TCCAGGCTTCAAGGAATGGAC |
|  | Reverse Primer | CAAGGCACGTTGCACGATG |
| *Bcat2* | Forward Primer | TGGAGTGGAATAACAAGGCTG |
|  | Reverse Primer | GTCTCCACCTTTGTATGCTTTC |
| *COX IV* | Forward Primer | ATTGGCAAGAGAGCCATTTCTAC |
|  | Reverse Primer | TGGGGAAAGCATAGTCTTCACT |
| *Cytochrome C* | Forward Primer | CCAAATCTCCACGGTCTGTTC |
|  | Reverse Primer | ATCAGGGTATCCTCTCCCCAG |
| *G0/S2* | Forward Primer | GTGAAGCTATACGTGCTGGG |
|  | Reverse Primer | CCGTCTCAACTAGGCCGAG |
| *KLF15* | Forward Primer | GAGACCTTCTCGTCACCGAAA |
|  | Reverse Primer | GCTGGAGACATCGCTGTCAT |
| *PGC-1* | Forward Primer | TATGGAGTGACATAGAGTGTGCT |
|  | Reverse Primer | CCACTTCAATCCACCCAGAAAG |
| *PRDM16* | Forward Primer | GACATTCCAATCCCACCAGA |
|  | Reverse Primer | CACCTCTGTATCCGTCAGCA |
| *PRODH* | Forward Primer | CGGTTCTTCCATCAAATGGCT |
|  | Reverse Primer | CATCTTTGCGATGCTGTCCTG |
| *UCP1* | Forward Primer | GGCCTCTACGACTCAGTCCA |
|  | Reverse Primer | TAAGCCGGCTGAGATCTTGT |
| *18s* | Forward Primer | CACACGCTGAGCCAGTCAGT |
|  | Reverse Primer | AGGTTTGTGATGCCCTTAGATGTC |
| **Human genes** |  |  |
| **Gene name** |  | **Sequence 5´to 3´** |
| *KLF15* | Forward Primer | TTCTCGTCGCCAAAATGCC |
|  | Reverse Primer | CCTGGGACAATAGGAAGTCCAA |
| *PRODH* | Forward Primer | CAGCCACATGGAGACATTCTTG |
|  | Reverse Primer | AGCCGTCATCGCTGACTCTAC |
| *18s* | Forward Primer | GGATGTAAAGGATGGAAAATACA |
