# Supplementary material for: Control of brown adipose tissue adaptation to nutrient stress by the activin receptor ALK7
Source: eLife. 2020 May 5;9:e54721. doi: 10.7554/eLife.54721 (PMC7200161; doi:10.7554/eLife.54721)
Supplement: Supplementary file 2. — The primary antibodies used in this study are listed here. [file elife-54721-supp2.docx]

**Supplementary Table 2: Antibodies**

| **Antibody** | **Reference** |
| --- | --- |
| Actin | Cell Signaling 4967 |
| Akt | Cell Signaling 9272 |
| ATGL | Cell Signaling 2138 |
| ATPB | Abcam 14730 |
| COX IV | Cell Signaling 4844 |
| HSL | Cell Signaling 4107 |
| mTOR | Cell Signaling 2972 |
| NDUFA10 | Santa Cruz 376357 |
| p-Akt_473_ | Cell Signaling 9271 |
| p-HSL_563_ | Cell Signaling 4139 |
| p-mTOR_2448_ | Cell Signaling 2974 |
| p-mTOR_2481_ | Cell Signaling 2971 |
| POX | Abcam 93210 |
| Rieske FeS | Santa Cruz 271609 |
| SDHA | Cell Signaling 5839 |
| UCP1 | Chemicon 1426 |
